# High flux water purification using aluminium hydroxide hydrate gels

**DOI:** 10.1038/s41598-017-17741-z

**Published:** 2017-12-12

**Authors:** Ali Malekizadeh, Peer M. Schenk

**Affiliations:** Food and Water Security Laboratory, School of Agriculture and Food Sciences, The University of Queensland, Brisbane, Queensland 4072 Australia

## Abstract

Filtration of aqueous liquids has wide implications, for example for provision of clean drinking water. Nevertheless, many people still lack access to safe water and suffer from preventable water-borne microbial diseases. This study reports a new ultrafiltration-range separation technology using a gelatinous layer of aluminium hydroxide polyhydrate as a secondary membrane on a retaining fabric that enables simple and cost-effective production of filtered water. Properties include at least 4-fold higher flux rates than currently available membranes, pressure-resistance, impenetrability to filtered particles, easy cleaning by backwashing and simple, cost-effective replacement by gel injection. Depending on the substrate, filtration is achieved through a packed bed of 1–2 nm hydrate gel globules, partly by mechanical straining with a size exclusion of approx. 10 nm and partly by physical adsorption. As a result, filtration of water (e.g. turbid river water) contaminated with colloids and microorganisms, including viruses, yields clear water that is free of measurable particles or detectable microorganisms. However, small water-soluble molecules (salts, sugars, proteins) remain in the filtrate. The findings demonstrate the potential for wide applicability of hydrate gels in high-flux and low-cost water purification devices.

## Introduction

Much progress has been made in the development of filtration and separation devices. These include microfilters, high-pressure membranes (e.g. ultrafilters, nanofilters, reverse osmosis, membrane bioreactors) for various applications, including water purification and the isolation of valuable compounds^1–5^. However, it is estimated that over 1 billion people still lack access to safe drinking water and often suffer from preventable water-borne protozoan, bacterial and viral diseases^6,7^. Robust and low-cost water filtration devices are still needed to make clean, safe water more affordable and address issues of pathogen contamination and environmental pollution^1,5^.

Drinking water is generally produced by a series of coagulation, flocculation, sedimentation, flotation, sand filtration and chlorine disinfection^8^. Pressure-driven membrane filtration for water treatment applications has gained in popularity based on advantages, including better and more consistent water quality supply, smaller system and reduced land requirements, and better control on operation^1,9–12^. Shrinking of high-quality water resources and stricter water quality regulations are other driving forces for using membranes rather than conventional water treatment processes^8,13^. However, this technology has not been widely used for large-scale water treatment (except for desalination)^12^; the main limitations are high energy consumption, elaborate membrane cleaning procedures and flux decreases due to membrane fouling^14^. Particulates, microorganisms, dissolved inorganics and natural organic matter are some of the causes of surface or internal membrane fouling for water treatment^15–17^. Ideally, the water feed to membranes should not have suspended solids, therefore any functional membrane water treatment plant requires adequate pre-treatment processes (e.g. coagulation, sedimentation and sand filtration)^18–21^.

Microfiltration (MF) has the largest pore size (0.1–10 µm) and highest permeability (>1000 L/h.m^2^.bar) and is generally used for clarification, pre-treatment and removal of bacteria based on screening or, more commonly, depth filtration. The lifetime of the filter depends on its application (e.g. several months for air vents or single use cartridges). Ultrafiltration (UF) employs smaller pore sizes (1–100 nm) and less permeability (10–1000 L/h.m^2^.bar) which also removes macromolecules and viruses. Nanofiltration (NF) has even lower pore sizes (0.5–2 nm) and very low permeability (1.5–30 L/h.m^2^.bar), also removing multivalent ions and some small organic compounds, color, hardness and a fraction of dissolved salts^15^. Nanofilters were developed to address high pressure, energy costs and low water permeability of reverse osmosis (RO) membranes^15^. RO has the smallest pore size (< 0.5 nm) and the lowest permeability (0.05–1.5 L/h.m^2^.bar) and is used for desalination to produce ultrapure water, removing monovalent ions.

The mechanisms of MF and UF separation is sieving, while NF involves sieving, charge effects and diffusion, and RO filters employ solution diffusion^1,22^. MF and UF are generally used for particle and microbial removal from water, while NF and RO also remove dissolved organic matter^13^. MF and UF filters can remove most bacteria and all protozoa when the membrane operates properly^13^. However, virus removal by membranes is very specific and highly related to the type of membranes and the virus. High virus rejection from water has been achieved by UF membranes and partial virus rejection by MF membranes^23^. For example, 0.22 µm Millipore cellulose nitrate membranes filters remove about 45% of poliovirus from tap water^24^. However these studies only examined short-term virus removal (e.g. 5 mL of virus suspension^25^ or 100 mL of virus suspension^23^). Another study on much smaller bacteriophages using different MF and UF membranes showed that microfilters with pore sizes of 0.2 and 0.1 micron can only remove MS2 bacteriophages with 0.2 and 0.3 logs, respectively, while nanofilters with pore sizes of 500 kD (20 nm), 300 kD, 100 kD removed MS2 bacteriophages with 1.7, 4 and 6.8 logs, respectively^13,26^. Microorganism rejection is also related to pressure and flux, as at high pressures and fluxes the rejection decreases^11,27^. Furthermore, some microorganisms can pass membranes with smaller pore sizes due to the existence of some openings larger than the nominal pore size of the membrane^11,28,29^.

Filtration can be greatly assisted by secondary or dynamic membranes that are positioned on top of a primary membrane. This layer may also prevent the primary membrane from fouling by removing finer particles^30,31^. Diatomaceous earth, perlite, and cellulose are some examples of these precoat filter aids. For example, microfiltration of activated sludge in wastewater treatment is assisted by kaolin, lime and diatomaceous earth as dynamic membranes to solve the issue of rapid flux decline, the main problem in microfiltration of activated sludge^32^. Moving filter cakes of cross-filter membranes that build up on top of primary membranes can also act as dynamic membranes^33^.

The present study developed a new secondary membrane, aluminium hydroxide polyhydrate, as a filter for aqueous liquids with potentially wide implications. To our knowledge, aluminium hydroxide hydrate gels that form from precipitates during their synthesis in water, although a well-known material, had not previously been used as separation devices. However dried crystalline metal oxides/hydroxides, such as aluminium hydroxide, are commonly used for flocculation and coating of sand filters to enhance the removal of bacteria by adsorption^34–36^. The present study shows that the mode of operation of an aluminium hydroxide polyhydrate filter placed onto a support mesh is potentially quite different to the operation of conventional ultrafiltration membranes.

## Results and Discussion

### Development of aluminium hydroxide hydrate gel as filtration material

Metal hydroxide hydrates comprise a number of water molecules that are attracted to the positive and negative charges of metal hydroxide and are trapped between scaffolds of hydroxide molecules when forming a gelatinous matrix^37^. Therefore, we hypothesized that water molecules can easily exchange through polyhydrate agglomerates of aluminium hydroxide hydrate when placed on a support mesh, allowing a flow of water.

To test whether aluminium hydroxide polyhydrate gel can be used as a filter for water-based liquids, aluminium hydroxide hydrate was produced *in situ* in an aqueous solution by mixing aluminium sulphate and sodium bicarbonate (baking soda) solutions (Reaction 1) or by electrolysis of saline water using aluminium electrodes and DC current. In both cases, aluminium hydroxide hydrate quickly formed leading to a gelatinous precipitate that could be placed onto a support mesh for filtration purposes. It was found that both, aluminium sulphate and sodium bicarbonate are required for hydrate filter formulation, as direct addition of pre-soaked aluminium hydroxide did not result in the formation of coherent gelatinous polyhydrate. Both salts are available at low cost and are not classified as harmful chemicals. To investigate whether there is a difference in how water molecules bind to dissolved crystalline Al(OH)_3_ compared to aluminium hydroxide hydrate gel, the water evaporation rate was measured. Supplementary Figure 1 shows that crystalline Al(OH)_3_ dissolved in hot or cold water, dried after 60 h, leaving Al(OH)_3_ powder, while aluminium hydroxide polyhydrate gel produced by chemical reaction *in-situ* only dried after 160 h, leaving a hard crystalline material. We hypothesized that Al(OH)_3_ molecules when formed in water are immediately surrounded by a varying number of water molecules attracted to the positive and negative charges via hydrogen bonds forming polyhydrate agglomerates. These then condense, partly by polymerization (forming small Al(OH)_3_ crystals of varying sizes) and partly by merging together to form larger aggregates that together appear as a gel-like amorphous structure.

1 $${{\rm{Al}}}_{2}{({{\rm{SO}}}_{4})}_{3}+6{{\rm{NaHCO}}}_{3}\to 3{{\rm{Na}}}_{2}{{\rm{SO}}}_{4}+2{\rm{Al}}{({\rm{OH}})}_{3}+6{{\rm{CO}}}_{2}$$

**Figure 1:**
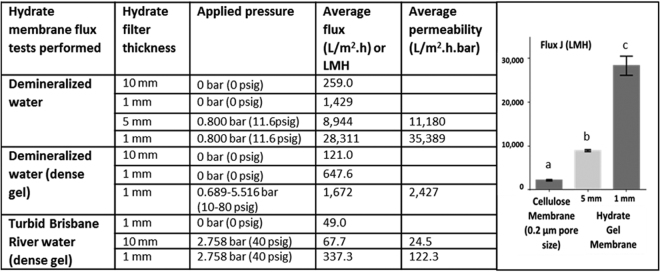
Use of aluminium hydroxide hydrate gels for water filtration and comparison to conventional MF. The values show examples of various hydrate gel filters (normal or denser material at different strengths) under various pressures with either demineralized water or highly contaminated river water. Different small letters indicate statistically significant differences (Student t-test; n = 3; *p* < 0.05). For a comprehensive table comparing hydrate gel filtration to a wide range of examples of MF, UF, NF and RO applications, see Supplementary Table 1.

Filtration tests were then carried out where the produced hydrate gel in suspension was placed on a holding fabric which upon settling formed a uniform gel layer with 10 mm thickness. Using this device, the unassisted base flux rate with demineralized water was 259 L/m^2^.h (LMH) at 20 °C and a head pressure of 10 cm (10 mbar) (Fig. 1). The flux rate increased to 1,429 LMH when using a thinner gel layer of 1 mm and this further increased to 28,811 LMH when applying pressure (0.8 bar). A detailed pressure-flux response curve with a hydrate gel (1 mm) showed that fluxes did not further increase at pressures above 0.689 bar (Supplementary Figure 2). To directly compare hydrate filtration to the performance of conventional membranes, a cellulose MF membrane (Mixed Cellulose Ester 0.2 µm, 25 mm diameter) was used under identical conditions using a 1 mm or 5 mm thick hydrate gel (25 mm diameter) with a cotton liner fabric as holding matrix and 0.8 bar applied pressure. The results (Fig. 1) showed that hydrate gel filtration (even with a layer of 5 mm) performed significantly (*p* < 0.05) faster than the cellulose membrane.

The observed mechanism of hydrate gel filtration was fundamentally different to gels from polymers; for example, a 2% agarose gel was unable to filter water (even after several days). A denser hydrate gel was produced by mixing stoichiometric supersaturated (by heating) solutions of aluminium sulphate and sodium bicarbonate at near 100 °C. We hypothesized that the presence of more aluminium hydroxide in a denser hydrate gel (Al(OH)_3_ × 37 H_2_O) would lead to stronger interactions with water molecules and stronger retention compared to hydrate produced at 20 °C (Al(OH)_3_ × 90 H_2_O). Indeed, when placed on a support mesh, the flow rate was less than halved (121 LMH) for denser hydrate gels (Fig. 1). Similarly, flux rates were also reduced at lower temperatures (4 °C).

### Filtration properties of different aluminium hydroxide polyhydrate gels

**Figure 2:**
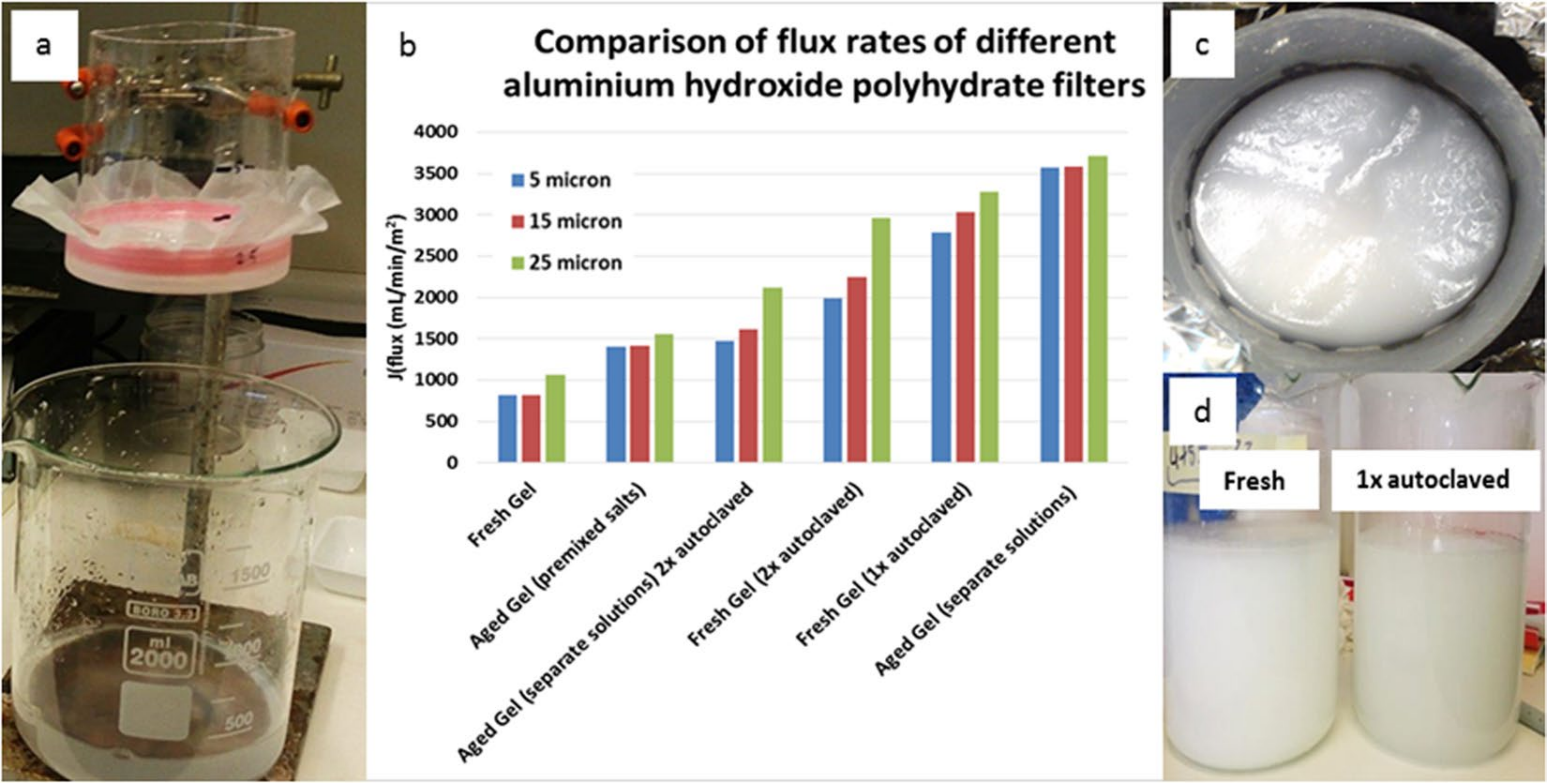
Comparison of flux rates of different aluminium hydroxide polyhydrate gels used for water filtration with holding meshes of various sizes. (**a**). Basic experimental setup. (**b**). Standard filtration conditions included the use of 100 mL of gel (10 mmol Al_2_(SO_4_)_3_, 64 mmol NaHCO_3_) with 300 mL of water and a head pressure of 5 cm or 5 mbar (0.073 psig). Three parameters were tested: reaction conditions (premixed aluminium sulphate/sodium bicarbonate salts vs. separate premade aluminium sulphate and sodium bicarbonate aqueous solutions), age (fresh (hours) vs aged (1 month)), and autoclaving (no vs. once or twice of autoclaving) for each of three holding fabrics (5, 15 and 25 micron). (**c**). Top view. (**d**). White aluminium hydroxide hydrate gel freshly prepared and after autoclaving (note the color change from white to a greyer, more gelatinous and transparent hydrate).

The hydrate gel forms a uniform layer on a filter-retaining material as long as the material is even and flat and the gel coat is of sufficient thickness. When the gel suspension was added to a flat porous layer of at least 25 µm pore size, gel particles settled evenly, forming a gel and a water layer. For example, a 1 mm thickness was found to evenly coat a 25 µm fabric or sintered titanium disc. To better understand how hydrate gel properties influence flux rates, various combinations of hydrate gel preparations were tested with different-size support matrices (Fig. 2). Hydrates of different formulas, concentrations, densities and thicknesses may also be used for potentially different applications. Adding the ingredients separately directly as salts to water (rather than as premixed solutions) also formed aluminium hydroxide polyhydrate, but the use of dissolved reagents was more efficient to rapidly form polyhydrate. When hydrate was produced by mixing aluminium sulphate and sodium bicarbonate solutions, the mixture appeared milky and did not show any initial signs of gelatinousness. However, as the mixture was allowed to settle and condense, a coherent gel formed within minutes which became denser and more gelatinous over time. This condensation process was accelerated by the purity of the water used, temperature, applied pressure, water contents and age of the gel, which was also accompanied by a color change from milky white to light grey/transparent (Fig. 2). For example, the gel condensed by 50% of its original volume within 7 days of its production and then further condensed at a much lower rate over several months.

The initial size of hydrate gel flocs from freshly-produced gel was between 5–15 µm in diameter. This was evident from various holding fabrics with pore sizes of 5, 15 and 25 µm that showed that only the screen fabric with 5 µm was able to immediately hold all freshly-produced aluminium hydroxide polyhydrate gel. As the gel aged over several days up to 3 months, it condensed further and was stably held in place by fabrics with much larger pore sizes (e.g. 0.5 mm). Gelatinousness (indicating coherence and condensation state of hydrate molecules) also further increased as fresh hydrate solution was washed to remove excess sodium sulphate produced by the reaction and the color changed from milky white to light grey. Sodium sulphate washout in the filtrate was reduced to 0.1 g/L after two washes (300 mL each) and was undetectable (gravimetrically) after three washes. Similarly, higher temperature and pressure of the mixture generated by autoclaving also increased the condensation state of hydrate molecules and the gelatinousness of the hydrate aggregates. After 20 min of autoclaving, the hydrate solution changed to a more gelatinous and transparent grey material. Condensation of the hydrate solution further increased by prolonged or repeated (3x) autoclaving. Interestingly, aged or (1x) autoclaved hydrate gel (but not both) formed a more consistent and more homogenous polyhydrate layer and displayed the highest flux rates among the conditions tested (Fig. 2), suggesting that aging at room temperature or short autoclaving may lead to a better alignment of the hydrate layer pores. However, repeated autoclaving reduced the flux rates. Hence, subsequent tests, unless otherwise stated, were performed on using aged gel. Preliminary rheology studies revealed that aluminium hydroxide polyhydrate gel (aged 74 days at room temperature) had a reversible structure which showed resistance to shear rate increases (until breaking of the structure), and reshaped its structure when it was stationary. These properties fit best to a Bingham plastic model which behaves as a rigid body at low stresses but flows as a viscous fluid at high stresses (e.g. similar to mayonnaise). In summary, hydrate gel, manufactured by mixing separate saturated aluminium sulphate and sodium bicarbonate solutions, that was aged for at least 1 month at room temperature and stored under water displayed the best performance.

Taken together, this supports the hypothesis that aluminium hydroxide molecules when synthesized in water, quickly attract water molecules, forming polyhydrate units that further condense. These aggregates become more structured over time or with heat, resulting in a denser, more gelatinous and more transparent material. It appears plausible that any aluminium hydroxide polyhydrate gel comprises a mixture of both, loosely and firmly condensed hydrate molecules, that also includes aluminium hydroxide polymers of varying lengths that may partially crystallize within the gel. Accordingly, properties change depending on how the gel was produced and then stored or treated. The increased transparency of more condensed material may indicate the presence of a regular structure that self-organizes based on positive and negative charges where water channels form that allow the use of this material as a filter with a defined pore size.

### Aluminium hydroxide polyhydrate filtration effectively removes colloids and contaminating microorganisms, including bacteriophages, from water

**Figure 3:**
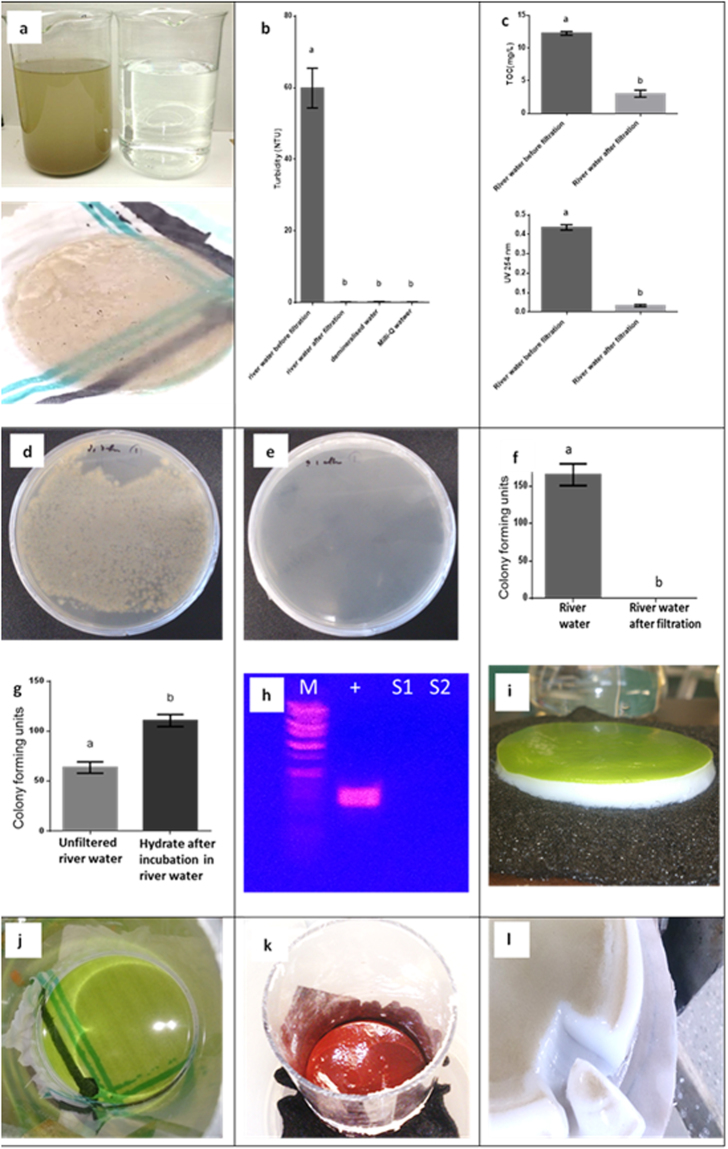
Various examples of hydrate filtration, including water quality and microbial contamination tests of Brisbane River water before and after hydrate filtration. (**a**). Turbid Brisbane River water before and after gravity-assisted hydrate filtration (top) and 3-fold autoclaved hydrate layer (bottom) used as filtration device (note the partial transparency that permits visibility of the holding fabric and increased flux rates). (**b**). After hydrate filtration, turbidity was significantly lower than river water before filtration [one-way ANOVA; *p* < 0.05] and showed no significant difference to demineralized water; NTU = Nephelometric Turbidity Units. (**c**). Significant decreases in total organic carbon (TOC; t-test; *p* < 0.05; top) and in UV 254 nm (Spectral Absorption Coefficient (SAC; t-test; *p* < 0.05; bottom) of river water after hydrate filtration. (**d**). LB plates with culturable bacteria from river water before filtration (average 2108 cfu/plate after 24 h of incubation; 5 plates were used). (**e**). LB plates from filtered river water after hydrate filtration (no bacterial colonies were found after 24 h of incubation). (**f**). Removal of bacteria by hydrate filtration. (**g**). Removal of bacteria by adsorption to aluminium hydroxide polyhydrate. (**h**). Detection of M13K07 Helper Phage DNA following hydrate filtration. M: wide-range DNA ladder (Takara). + : PCR-amplified phage DNA fragments (positive control); S1, S2: PCR reactions of filtrate samples. (**i**). *Scenedesmus dimorphus* NT8c microalgae harvested in the filter (also shows the self-supporting nature of the aluminium hydroxide hydrate gel). (**j**). *S. dimorphus* NT8c after filtration with a 3-fold autoclaved hydrate gel (note the transparency). (**k**). *Haematococcus pluvialis* UQ1 microalgae were harvested (source of the red pigment and strong antioxidant, astaxanthin). l. 10 mm hydrate layer at the end of river water filtration under applied pressure (5,516 mbar (80 psig)).

To test filtration properties of water with high colloidal contents, highly turbid river water with an optical density (OD) at 450 nm of 0.2 and significant amounts of suspended and colloidal solids was used (Fig. 3; Supplementary Figure 3a). River water passed through a 1 mm hydrate layer at a flux rate of 49.0 LMH without application of external pressure (10 mbar head pressure; Fig. 1; Fig. 3a). Pressure-assisted filtration of turbid river water through 1 mm and 10 mm hydrate filters, led to average flux rates of 337.3 LMH and 67.7 LMH, respectively (Fig. 1; Fig. 3). In all cases, the filtrate was clear and indistinguishable from demineralized water by OD measurements (OD^450 nm^ = 0.000) and light microscopy, suggesting that OD-detectable suspended solids and fine particles were effectively trapped on top of the layer of aluminium hydroxide hydrate (Fig. 3a). Next, different standard water quality tests were performed on river water filtrate (Fig. 3b,c). The results confirmed that the turbidity of filtrate was indistinguishable from demineralized water. Hydrate filtration also significantly decreased Total Organic Carbon (TOC), and the Spectral Absorption Coefficient (SAC); UV 245 nm absorption was used as a measure for organic compounds.

To test whether the use of clean water is a prerequisite for the generation of aluminium hydroxide polyhydrate, the ingredients Al_2_(SO_4_)_3_ and NaHCO_3_ were directly added to Brisbane River water (OD^450 nm^ = 0.145) as salts (separately or in a premixed 4:5 (v/v) ratio). In both cases, polyhydrate aggregates formation was unaffected, allowing immediate filtration. Although some colloids were visible inside the gel matrix, the OD after filtration was indistinguishable from demineralized water by optical density (OD^450 nm^ = 0.000).

For surface water treatment (lakes, rivers, reservoirs), coagulation, flocculation/sedimentation and conventional filtration processes mainly remove debris, larger particles and suspended solids, but still require disinfection by chlorine, to date the single most-effective measure to sanitize water^38,39^. However, concerns have been voiced over chlorine-resistant water-borne pathogens and chlorine disinfection by-products with various negative effects on human health chemical reactions within the water distribution network^5,40^. To test whether hydrate filtration is suitable to produce safe water and may assist conventional water treatment to remove microorganisms, we performed several, protozoan, fungal, bacterial and viral tests on filtrate recovered from hydrate-filtered river water. Tests with two highly visible microalgal species, *Scenedesmus dimorphus* NT8c and *Haematococcus pluvialis* (0.5 g dry weight/L) demonstrated that microalgae were effectively captured by hydrate filtration (Fig. 3i–k). Next, bacterial and fungal cultivation tests (five replicates per test) were performed on filtrate after hydrate filtration. All test results showed no bacterial and fungal growth from filtrate water (Fig. 3d–f; Supplementary Figure 3b,c), while unfiltered water displayed 2 × 10^4^ bacteria cfu/mL and some fungal colonies after 24 h and 1 week of cultivation, respectively, indicating that at least 99.99% of culturable bacteria and that fungi can be removed by hydrate filtration. The use of electrolysis-prepared aluminium hydroxide hydrate showed the same result. To rule out that aluminium hydroxide hydrate directly inactivated the bacteria, river water was mixed with aluminium hydroxide hydrate, but bacterial growth remained unaffected.

To better understand the mechanism of filtration, we examined whether filtration of bacteria by aluminium hydroxide polyhydrate was based on adsorption or filter pore size, or both. For this purpose in a separate experiment, 200 µL polyhydrate aggregates were incubated with Brisbane River water for 10 s or 10 min and compared to the same volume of river water without hydrate addition. The hydrate aggregate contained approx. 50% higher bacterial loads (shown as colony forming units (cfu) in Fig. 3g) compared to the same volume of river water, explaining that adsorption of bacteria to aluminium hydroxide hydrate occurred. However, deeper layers of aluminium hydroxide polyhydrate gel did not display any detectable bacteria by microscopy or plate cultivation, suggesting that both, mechanical straining and physical adsorption, are features of polyhydrate filtration.

This being the case, it was interesting to test whether a continuous reuse of impure hydrate gel was possible. After initial filtration with dirty river water, a stress test was performed by vigorously mixing the accumulated particles in the retentate after filtration with hydrate gel followed by repeated reuse of the dirty brown gel for river water filtration. For the first three times of mixing the gel with retentate matter, no sign of bacterial growth after filtration was observed and the filtrate was completely clear with no sign of suspended solids (OD^450 nm^ = 0.000). However, after mixing the filtrate with gel for the fourth interval, some bacterial growth was observed from filtrate. At this stage, 99.5% bacteria were still removed by the gel and the filtrate was still clear (OD^450 nm^ = 0.000). This shows that as the ratio of gel to dirt and contaminants decreases, some bacteria are able to pass the gel layer. Although the gel also absorbs bacteria, it is advisable that after repeated heavy load use, the retentate should not be mixed with the gel layer and it would be advisable to provide a protective mesh on top of the gel filter to enable clean removal of retentate to minimize or avoid disturbance with the gel layer.

To quantify the bacteria removal rate under extreme heavy microbial contamination conditions, a saturated freshly-grown *E. coli* culture was directly used for hydrate filtration. At the end of the filtration process a thick layer of *E. coli* bacterial biomass built up on top of the gel, demonstrating the strong mechanical straining properties of the gel. However, under these extreme conditions a small number of bacteria were able to pass through the gel. The bacterial removal rate for hydrate gel was 2 × 10^−7^ (> 99.9999%). While these high bacterial loads would not occur outside the laboratory, this test demonstrates that hydrate gel filtration can also perform under conditions of extreme bacterial contamination.

Enteric viruses are a major cause of human water-borne and water-related diseases and even bacteriophages, such as cholera-toxin inducing bacteriophages are of major concern, as they cannot be easily removed by filtration^41^. To test whether hydrate filtration is suitable to remove viruses from water, spiking of water with M13K07 helper phages (3 × 10^8^ plaque forming units (pfu)) was carried out followed by hydrate filtration. Contrary to the positive control, PCR of the filtrate water could not detect any phage DNA, suggesting that hydrate filtration effectively removed at least 99.99% of detectable viruses, including bacteriophages from water (Fig. 1h; Supplementary Figure 3d,e). To test whether water with even smaller bacteriophages can be decontaminated, MS2, a spherical bacteriophage of about 20 nm diameter was used. The results show that all plaque forming units (pfu) were removed by the filter, demonstrating that the filter can remove at least 99.99999% of MS2 phage viruses (log 7 scale).

To determine the lower limit of particles that can be removed by hydrate filtration, even smaller particles were used. We noted previously that small water-soluble molecules, such as salts and sugars readily passed aluminium hydroxide hydrate filters. To extend tests towards larger water-soluble molecules, such as proteins, bovine serum albumin (BSA; MW = 66.5 kDa; hydrodynamic diameter = approx. 7 nm) was used. The filtrate was yellow containing BSA protein, suggesting that the filter size exclusion limit lies between 7–20 nm and that water-soluble proteins of 7 nm hydrodynamic diameter or smaller cannot be separated from water by hydrate filtration, although this may differ for more hydrophobic proteins.

### Mechanism of hydrate gel filtration

Secondary or dynamic membranes typically make use of porous solid materials or porous filter cakes. Hydrate gel membranes may also be classified as secondary or dynamic membranes as the material is placed onto a primary membrane that acts as retaining fabric. To our knowledge, no research has been carried out on using polyhydrate gels as a secondary membrane. The mechanism of hydrate gel filtration also differs substantially from conventional solid precoat filter aids (e.g. diatomaceous earth, kaolin, lime) that do not reject hydrophobic compounds or filter nano-sized materials, such as viruses.

**Figure 4:**
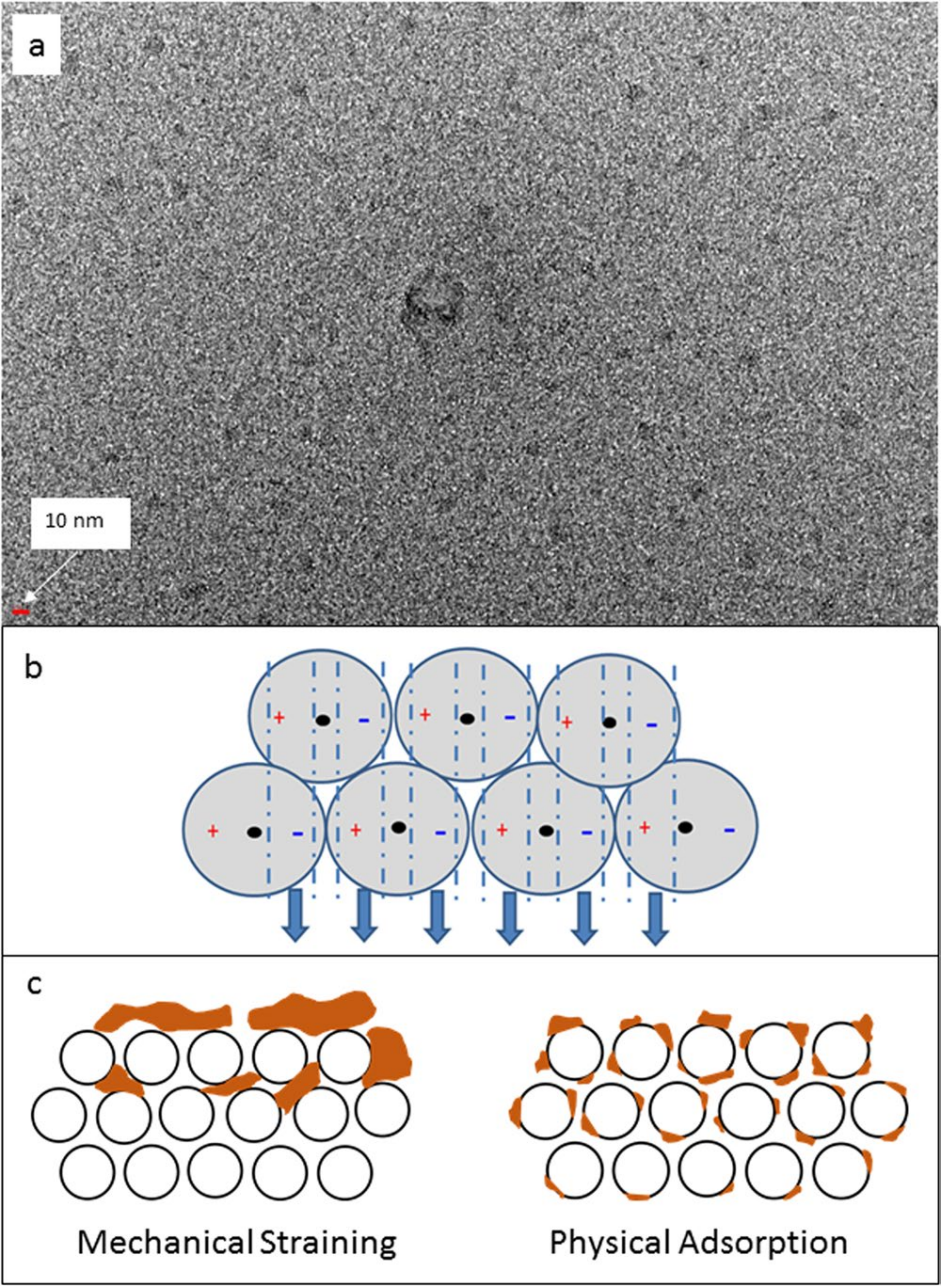
Material characterisation and model of hydrate filtration functionality. (**a**). Silicon Nitride TEM window of wet hydrate gel by SiMPore (product number, SN100.AZ0Q33); (**b**). Model of the formation and potential mode of action of an aluminium hydroxide polyhydrate filter. (**c**). Model of hydrate gel filtration mechanism where small (1–2 nm) hydrate globules form a packed bed. The results suggest that packed bed separation using hydrate gels involves both, mechanical straining (surface and depth straining) and physical adsorption.

To better understand the molecular mechanism of aluminium hydroxide polyhydrate filtration, X-ray powder diffraction and microanalyses by electron microscopy were performed. XRD analyses suggested that dried material included dawsonite, NaAlCO_3_(OH)_2_ (Supplementary Figure 4a). As we hypothesized that hydrate filtration has an underlying matrix that harbors water molecules, sequential dewatering was carried out to reveal the presence of an underlying structure. This resulted in the formation of a light-weight aerogel (Supplementary Figure 4b). Scanning and Transmission Electron Microscopy (TEM) analyses revealed a series of irregularly shaped crystalline fibres. However, it was realized that properties of this dried material would be far from those of a wet polyhydrate gel. Hence, the new technique of silicon nitride TEM window was employed where wet gel material can be trapped and analysed by electron microscopy. TEM window analyses revealed the presence of small 1–2 nm globules of regular size but that were arranged in an irregular manner (Fig. 4a). The exact mechanism of filtration of this layer is not fully revealed by these images, but it appears that hydrate gel may behave like a viscous liquid or packed bed, where particles may accumulate of top of the layer by mechanical straining. However, as the hydrate layer contains a large amount of water (typically 90 molecules for each Al(OH)_3_ produced), materials that are small and hydrophilic would freely pass through the gel layer, while hydrophobic compounds (irrespective of size) and larger molecules cannot pass this layer. This is consistent with the results of this study. As the small polyhydrate globules are charged (polar), they may form a more structured pattern over time or when heated (Fig. 4b). This is consistent with our observation that denser hydrates form with aging and/or heating which also become more transparent (Fig. 2). This basic structuring may then lead to increased flux rates (Fig. 2), but higher density gels (e.g. after repeat autoclaving) have led to reduced flux rates, most likely as this results in the presence of less water in the gel.

Data from this study also showed that physical adsorption contributes to the filtration process (Fig. 3g; Fig. 4c). TEM window images suggest that gel globules are arranged in a packed bed similar to sand filtration. Hence, it can be hypothesized that some particles may also enter deeper layers of the hydrate gel by displacing hydrate globules, but this process may be extremely slow and would be counteracted by the gelatinous structure of the hydrate filter layer. Previous tests have shown that visually all particles clearly form a separate layer on top of the gel during filtration. However, stress tests with high particle loads over long time periods may reveal if slow migration of particles through the gel layer are possible. More TEM window analyses of wet hydrate gels (e.g. examination of different layers after filtration) will be helpful for these studies.

### Potential uses of polyhydrate filtration

Collectively, the results from the present study show that not only bacteria, fungi and larger microorganisms, but also small viruses of approx. 20 nm (such as bacteriophages) can be removed from water using hydrate filtration. Assuming that human pathogens would also be captured (e.g. cholera-toxin inducing bacteriophages), this would result in filtrate water that is sanitized and significantly safer to consume. Hence this technology has potential to be used as a simple one-step water treatment system that removes suspended solids and pathogenic microorganisms, and may find applications in developing countries in areas with inadequate or no water treatment facilities. As the hydrate gel can be fitted into most shapes, there is potential to develop a variety of simple filtration devices. For example, plastic bottles may be fitted with hydrate filter lids or for larger volumes, perforated buckets with aluminium hydroxide hydrate gel can be used (Supplementary Figure 5a). Hydrate filtration may find applications in combination with conventional chlorine treatments in larger scale water treatment plants or as pre-filter for RO applications to reduce membrane fouling and associated expensive cleaning processes. Aluminium hydroxide is already widely used as a flocculant in water purification and is not considered toxic unless consumed in excessive amounts^36^. The material was tested and found stable under highly alkaline conditions (pH 13) but it should be considered that free aluminium ions may form when using highly acidic (<pH 5) liquids. Aluminium hydroxide hydrate filtration is also unable to remove water-soluble micropollutants, including heavy metal ions and hydrophilic pesticides and pharmaceuticals. Coupling of hydrate filtration with activated carbon or porous β-cyclodextrin polymer^38,42^ may provide a rapid and low-cost approach for safe water supply in the future. For example, the material costs required for water purification using aluminium hydroxide polyhydrate filtration when using a small-scale filtration device for at least 1,000 L of polluted water comparable to Brisbane River properties, are approx. US$0.003/kL (based on typical yields for the manufacturing of 50 mL gel, produced from 1.83 g Al_2_(SO_4_)_3_ and 2.66 g NaHCO_3_). By comparison, most filtration devices incur a cost of at least US$0.025/L. The actual costs will depend on how often the gel would need replacing which depends on the pollution grade of the water and whether a cross-flow design rather than dead-end can be used (see below). Indeed, one of the advantages of hydrate filtration is that hydrate gels can simply be removed by flushing and replaced by injection of new gel into the stream with potential for automation. If the gel is replaced rather than back-flushed, aluminium hydroxide will occur as a potential waste product. For a typical hydrate gel with approx. 2% aluminium hydroxide content, 1 g of waste product would occur for 1000 L of purified water (based on the example above). While aluminium hydroxide is considered non-toxic and usually inert at normal pH, it can also be converted into aluminium sulphate again by using sulphuric acid.

To test whether hydrate filtration can be permanently installed to household water supply, two prototypes were developed (Supplementary Figure 5d,e). The first prototype includes an inverted glass jar fitted with a water inlet and a 2 mm hydrate filter on a sintered titanium disc that was placed inside another sealed glass jar with a water outlet. The device was connected to a water reservoir positioned 1 m above the filtration unit to create a small head pressure. The water reservoir was connected to the mains water supply by a floating valve. It was able to produce 290 mL/min of filtered water and would be suitable to produce drinking water on a day-to-day basis from a small water reservoir (e.g. 10 L container or small rainwater tank). The second prototype used 150 mm PVC pipes with essentially the same design but at larger scale using a perforated plastic holding disc and a 2 mm hydrate layer and with direct connection to the mains water supply with a floating valve. This device was able to produce 1.58 L/min of filtered water and would be suitable to produce drinking water on a day-to-day basis for a family or small community from a large water reservoir (e.g. rainwater tank, dam or mains water supply that requires additional filtration). Both prototypes were operated for several weeks without disruption, which resulted in the built up of significant amounts of visible particles that may originate from small colloids from the water source or from particles that accumulated inside pipes over time.

### Adaptations of aluminium hydroxide hydrate filters for industrial purposes

For industrial applications, fast, cost-effective and easy maintenance filtration processes are desirable. Flux rates up to 28,000 LMH and permeabilities up to 35,000 L m^−2^.h^−1^.bar^−1^ were achieved with aluminium hydroxide polyhydrate gel filtration (Fig. 1). There are four main types of membranes in the market, microfilters, ultrafilters, nanofilters and reverse osmosis. Typical working flux ranges are between 10–30 LMH  and permeabilities range from 0.05–1.5 L m^−2^.h^−1^.bar^−1^ for reverse osmosis to 1.5–30 L m^−2^.h^−1^.bar^−1^ for nanofiltration, 10–1000 L m^−2^.h^−1^.bar^−1^ for ultrafiltration to > 1000 L m^−2^.h^−1^.bar^−1^ for microfiltration^1,5^. Hence hydrate filtration has the potential to perform significantly faster. Assuming a pore size of 10 nm, a comparison to silicon carbide ceramic membranes, which are considered to be one of the fastest commercially available membranes (Manufacturer’s specifications, LiqTech International, Ballerup, Denmark), shows that hydrate filtration has the potential to achieve approximately 100 times higher permeability rates. Supplementary Table 1 shows a comparison of the calculated average fluxes of hydrate filtration to conventional membranes, suggesting that even a hydrate filter at very low or no added pressure could potentially outperform conventional high pressure membranes.

In principle, a hydrate filter can be built in a variety of shapes as long as all the liquid passes through the hydrate layer. It can be adapted as a secondary membrane onto existing filters to enhance their properties and prevent fouling or simply used in combination with a simple retaining fabric. Supplementary Figure 6 depicts possible designs for various water filtration purposes. One concern for large-scale use of hydrate filters is that the hydrate is not strong enough to resist harsh filling. However our experiments showed that water accumulated on top of the hydrate can act as a protecting shield of the hydrate layer. Another, related concern is that hydrate filters can potentially dry out, relinquishing its filtration capability. A potential solution to overcome both issues could be by maintaining the gel under water (Supplementary Figure 6e) or by simply adding a layer of paraffin on top of the filter. The basic hydrate filtration unit comprises a holding filter medium (e.g. fabric, filter paper) that has a pore size that is sufficiently small to retain the hydrate gel. The hydrate layer is held on top of the holding fabric (at the desired thickness and density). Optionally, a fabric may be positioned on top the hydrate layer if particles or other components separated from a mixture are to be recovered (Supplementary Figure 6a). A top holding fabric is also required to enable cleaning of the filter to prevent blinding, e.g. by side- or back-washing (similar to sand filters). To test whether back-washing is an adequate method to remove accumulated particles from hydrate filters, a wide plexiglass tube with a holding fabric was used for Brisbane River water filtration (as described above) and a small pressure (3 mbar) was applied by placing it in surrounding water with 3 cm depth. All visible particles were lifted from the hydrate filter without disturbing the gel, effectively washing and clearing the hydrate filter. This would also allow easy recovery of particles (e.g. coal dust or other valuable particles from mining operations, as an alternative to flotation).

Flow configuration of membranes is either crossflow (tangential) or dead-end^39,43^. In a crossflow design, feed flow travels tangentially across the surface of the filter, rather than into the filter. Cross flow filters are more expensive but are more popular because they have less fouling issues and can work continuously. A pump can recycle and recirculate the feed several times around the unit until the desired concentration is achieved and the concentrated retentate is transferred out of the unit^43^. A basic possible design of a cross flow hydrate membrane is shown in Supplementary Figure 6b. Hydrate membranes can also be designed similar to conventional RO membranes (Supplementary Figure 6c). In this design water molecules are on both sides of the hydrate layer, and all sections of the hydrate layer are completely immersed in water at any time. This can be an advantage in some applications compared to previous designs. Given that a functional hydrate filter can form spontaneously and in impure water, a fully automated application can be envisaged. In this design, water passes through a holding fabric and the hydrate filter ingredients are simply injected as salts or as solutions, leading to rapid aggregation of aluminium hydroxide hydrate and gel formation. Once flow is reduced due to accumulation of particles, the hydrate filter can be simply flushed sideways, back-washed and/or replaced by a renewed injection of hydrate-forming ingredients.

### Conclusion

- Hydrate gel filtration can be used as a secondary membrane on a retaining fabric. It makes use of a simple aluminium hydroxide hydrate gel that enables water purification for removal of turbidity-causing pollutants and microbial contaminants (including bacteria, fungi, microalgae and viruses).
- Flux rates were up to 28,000 LMH with applied pressure (0.8 bar) and approximately 100-fold higher than currently available silicon carbide ceramic membranes.
- Aluminium hydroxide hydrate filtration has potentially broad significance for safe water supply and human health.
- Its low cost and adaptability to various shapes makes it attractive for developing countries and for assisting existing technology for a range of water treatment and purification applications.

## Materials and Methods

### Small-scale filtration test equipment

A wide plexiglass tube (with a diameter of 76 mm) with an open top and an open bottom was positioned such that a geotextile fabric (polypropylene 260 gsm nonwoven geotextile with a pore size of 90 micron) extended across the open bottom. Other holding fabrics were also used, based upon the specific application design, flow rates and the required strength of the retaining fabric, to support the hydrate layer. These included cotton fabric linen (most commonly used fabric), lightweight low-pore polyester fabric (Rip Curl boardshorts), and screen fabrics (5, 15, 25 micron pore size, Sefar, Heiden, Switzerland). In this manner, the layer of metal hydroxide hydrate was located at the bottom of the plexiglass housing. A collection beaker was positioned underneath the holding fabric to collect any liquid that passed through the layer of metal hydroxide hydrate. The thickness of the hydrate gel was 1–10 mm and the test liquid used was typically 100 mL. This was the filtration test equipment used in all methods unless mentioned otherwise (e.g. for pressure filtration). To compare to conventional membranes, demineralized water filtration of hydrate gel membrane was compared to a conventional cellulose membrane (Mixed Cellulose Ester 0.2 µm, 25 mm diameter, Avantec Inc). A total of 50 mL of demineralized water was filtered side-by-side by both filters. A total of 5 mL of 6 month-old hydrate gel was placed into a 50 mL syringe fitted with cotton as a holding fabric. After adding 50 mL of water, the water-gel mixture was left for 10 min to allow complete gel settling, resulting in 1–5 mm gel thickness. As head pressure was not sufficient for cellulose membrane filtration, 0.798 bar (11.58 psig) pressure was applied to both setups using a 4 kg weight placed on top of the 50 mL syringes.

### Pressure-assisted hydrate filtration

For mid-scale, pressure-assisted and unassisted hydrate filtration tests, a mining processing laboratory pressure filter made under AS 1210 class 3 standards by Amdel Company was used (Supplementary Figure 2a,b). The applied pressure was adjustable up to 6,895 mbar (100 psig). A low pore fabric (lightweight polyester; Rip Curl boardshorts) was placed at the bottom of the filter. A 2 micron pore size filter paper (Macherey Nagel, MN1640D, 185 mm diameter) was also placed on top of the fabric to retain the hydrate under pressure. The diameter and cross section area of this equipment were 0.155 m and 0.01887 m^2^, respectively. For each experiment, first hydrate solution (for example 50 mL to achieve 1 mm gel thickness) was added then after the hydrate particles settled naturally or by using pressure, the feed was added. After this the lid was closed and compressor-generated air pressure was applied and adjusted. The filtrate was collected in a beaker and weighed over time to measure the flow rate. Alternatively, for a more robust and permanent setup, a sintered titanium disc (135 mm diameter; 2 micron pore size; Baoji Qixin Titanium Co Ltd) was used as the gel holding matrix. This device was also used for pressure-assisted river water filtration. After the hydrate layer formed, the feed (1.8 L of Brisbane River water) was added and filtered under various applied pressures.

### Calculating pressure flux curves

For each experiment, first 100 mL of dense hydrate solution (prepared at 100 °C) was added to 2 L of demineralized water. This solution was added to the pressure filter equipment and the hydrate particles were allowed to settle for 10 min, forming a consistent hydrate layer with a thickness of either 1 mm or 10 mm. After carrying out filtration at different pressures, the filtrates were collected in a beaker and weighed over time. Based on these data the fluxes were calculated. During each filtration test, the flux was calculated eleven times in 2 min periods.

### Preparation and characterization of aluminium hydroxide polyhydrates

#### Aluminium hydroxide hydrate synthesis by electrolysis

Electrolysis-based aluminium hydroxide hydrate was produced by electrolysis of saline water (salinity ranged from 30 to 70 parts per thousand, PPT) using aluminium electrodes and DC current. A DC voltage from 3 to 12 V (preferably 9 V) and a current of 1 Amp was used.

#### Preparation of aluminium hydroxide hydrates with different densities

The reaction forming aluminium hydroxide hydrates is based on the following equation.

$${{\rm{Al}}}_{2}{({{\rm{SO}}}_{4})}_{3}+6{{\rm{NaHCO}}}_{3}\to 3{{\rm{Na}}}_{2}{{\rm{SO}}}_{4}+2{\rm{Al}}{({\rm{OH}})}_{3}+6{{\rm{CO}}}_{2}$$

As the solubility of each ingredient in water varies by changing temperature and pressure; based on solubility curves of each ingredient, the stoichiometrically saturated solutions of the ingredients for each temperature and pressure can be determined. A normal (soft) hydrate gel was produced by mixing stoichiometric solutions of aluminium sulphate and sodium bicarbonate at 20 °C. A dense hydrate gel was produced by mixing stoichiometric solutions of aluminium sulphate and sodium bicarbonate at near 100 °C. The hydrates were always washed for removing sodium sulphate made by the reaction. The hydrates were also left for several hours to allow all CO_2_ molecules to separate. At 20 °C, the solubility of aluminium sulphate and sodium bicarbonate in 100 mL of water are 36.4 g and 1.6 g, respectively. Therefore, at 20 °C, to make a stoichiometric mixture for use in the above equation with saturated solutions, 36.4 g of aluminium sulphate was dissolved in 100 mL water and 53.76 g of sodium bicarbonate was mixed with 560 mL water. Adding the aluminium sulphate solution to the sodium bicarbonate solution resulted in a stoichiometric reaction mixture to prevent built-up of excess aluminium sulphate or sodium bicarbonate at the end of the reaction. We also prepared a denser aluminium hydroxide hydrate by mixing the reactants in water at higher temperatures. For example, at 100 °C, the solubility of aluminium sulphate and sodium bicarbonate in 100 mL of water increases to 89 g and 23.6 g, respectively. Therefore, at 100 °C, to prepare a stoichiometric mixture for use in the above equation, 89 g of aluminium sulphate were mixed with 100 mL of hot water and 130.98 g of sodium bicarbonate were dissolved in 555 mL of hot water (near 100 °C). When these solutions were mixed, a thicker aluminium hydroxide hydrate was formed. Mixing the ingredients in a pressurized heated reactor (e.g. pressure cooker/autoclave) further increased the solubility of the ingredients to produce a very dense and highly charged aluminium hydroxide hydrate gel.

#### Comparison of filtration properties of different aluminium hydroxide polyhydrate gels

Different formulations of aluminium hydroxide hydrate gel were tested and compared to each other using the wide plexiglass tube (with a diameter of 76 mm) filtration equipment (explained above) with 5, 15, 25 micron pore size screen fabrics (Sefar, Heiden Switzerland). All gel samples were manufactured at room temperature using stoichiometric amounts of aluminium sulphate and sodium bicarbonate according to equation (1). Hydrate gel was either produced by mixing separate saturated solutions or by premixing the dry powders of the two salts before adding water. The different gel samples prepared from saturated solutions included: (1) fresh hydrate gel, (2) 1 x autoclaved gel (121 °C, 122 kpa, 20 min), (3) 2 x autoclaved gel (with cooling to room temperature between autoclaving). (4) one month-old gel (aged at room temperature), and (5) one month-old gel which was autoclaved 2 times. In addition, one gel sample was prepared from premixed powders that was also aged for 1 month. For each test, 100 mL of gel was mixed with 300 mL of distilled water and the mixture was added to plexiglass tubes and the filtrate was collected in a beaker to measure and compare the flux of different gels using different fabrics. To compare how different pore sizes of the fabric hold gel particles, optical densities (OD) at 450 nm of the filtrates were compared.

#### Production of thin layers of the hydrate filter

The bottom part of the Amdel pressure filter unit that was used for previous pressure filtration tests was upgraded using a flat sintered titanium filter disk with under 2 microns pore size. It was sealed using waterproof sealing glue (other sealing equipment such as rubber could also be used). The sintered titanium fixed on the bottom of pressure filtration equipment allowed the water to pass while the hydrate built a thin layer on top. Other similar sintered filter holders such as porous plastics, or fine fabrics, membranes or filter paper can also be used. Sintered titanium was found ideal at it is very durable and resistant to high pressure. It also has a very smooth shape that allows a very thin (0.5 mm) and consistent layer of hydrate to be built above. It is also acid and alkali resistant. Therefore, it is possible to easily acid wash it at the end of filtration to avoid accumulation of any deposits inside the filter. Low dosages of hydrate were mixed in water to make a very dilute hydrate solution. The dilute hydrate solution was added on top of the filter holder and allowed to settle. If the solution is very dilute the hydrate may not settle naturally. Therefore, low pressures (689–2,756 mbar (10–40 psig)) were applied for several minutes after pouring the dilute hydrate solution. This is a key step in the process which causes the hydrate in the solution to settle, forming a very thin consistent hydrate layer film on the retaining filter holder. After the thin hydrate layer was built, the feed was added and filtered.

#### Ratio of water molecules to aluminium hydroxide in hydrates

Hydrate forming solutions were poured into the filtration equipment as explained above, and after 1 h and removal of excess water, different hydrate preparations were collected and weighted. The number of water molecules and aluminium hydroxide molecules of the different hydrate formulas was determined by weighing different hydrate samples (5 replicates) on fabric media before and after freeze-drying.

#### Assessing the evaporation rate of various aluminium hydroxide hydrate gels

Different formulas of hydrate gels (dilute prepared at 20 °C or dense prepared at 100 °C by chemical reaction) and two aluminium hydroxide powder preparations were used. Aluminium hydroxide hydrate gel was produced at 20 °C or 100 °C as described above. Alternatively aluminium hydroxide powder (Sigma) was either soaked in cold water overnight or mixed in hot water (near 100 °C) for 2 h where aluminium hydroxide molecules absorbed some water molecules forming a material resembling a hydrate. To test water evaporation rates of different hydrates, they were first poured on top of the filtration equipment used for previous tests and the hydrate was freed of excess water and salts by water flow for about 1 h. Three replicates of each water-drained hydrate (approx. 2.5 mL each sample) were then spread evenly into 15 mL centrifuge tube lids. The samples were allowed to dry at room temperature and were weighted and plotted over time.

#### Production of aerogel from aluminium hydroxide hydrate

A total of 5 mL of different gel samples was washed by absolute ethanol in a Falcon tube fitted with a Taffeta fabric at the bottom for one week. This process removed more water molecules than just by soaking, as gravity also facilitates alcohol-water molecule interactions. Filtrate conductivity measurement was used as a simple indicator of water removal. After water molecules were replaced with ethanol, the samples were placed inside an automated tousimis Autosamdri®-815, Series B supercritical dyer. This equipment replaced the ethanol molecules with supercritical CO_2_. Because supercritical CO_2_ behaves like gas, no structure shrinkage occurred that otherwise appears due to surface tension of liquids during normal drying. Therefore no collapsing occurred and the structure was maintained. To enable the manufacturing of an ideal aerogel, the equipment was run in manual mode for 3 h with repeated manual filling and purging periods every 30 min.

#### X-ray powder diffraction

X-ray powder diffraction (XRD) is a rapid analytical technique primarily used for phase identification of a crystalline material that can provide information on unit cell dimensions. Hydrate gel samples were oven-dried overnight (60 °C). The analysed material was finely ground, homogenized, and its average bulk composition was determined before being subjected to XRD analysis using a Bruker D8 Advance MkIII XRD unit. X-rays were shot at an angle to the solid, and the diffracted intensity was plotted against 2 theta, where theta is the angle of incidence of the x-rays.

#### Scanning and Transmission Electron Microscopy

Scanning and Transmission Electron Microscopy analyses were carried out at the Centre for Microscopy and Microanalysis (CMM) of the University of Queensland using a Joel 7001 instrument operating a 15 keV or 10 keV and a Joel 2100 electron microscope operating at 200 keV, respectively. Silicon Nitride TEM window of wet hydrate gel was performed by placing approx. 0.1 µL of hydrate gel onto grids by SiMPore (product number, SN100.AZ0Q33) using a Joel 2100 electron microscope operating at 200 keV.

### Applied hydrate gel filtration tests

#### Bacterial and fungal contamination tests

Lennox Lubert Berthani (LB) (10 g/L tryptone, 5 g/L yeast extract, 15 g/L agar, 10 g/L NaCl) or Potato Dextrose Agar (PDA; 4 g/L potato extract (from infusion of 200 g potato), 20 g/L dextrose and 15 g/L agar) medium was used for bacterial cultivation (LB) or a mixture of bacteria and fungi (PDA). The same amount of 20 mL of electrolysis-prepared aluminium hydroxide hydrate and chemical reaction-based aluminium hydroxide hydrate was used for each experiment. Turbid Brisbane River water was collected close to the University of Queensland, St Lucia campus, Australia. All filtration equipment was autoclaved and cooled before the tests. To avoid cross contamination, all tests were performed under a laminar flow. A layer of aluminium hydroxide hydrate was added on top of the holding fabric and 200 mL of Brisbane River water was added on top of the hydrate gel. The first 50 mL of the filtrate were not collected to allow for removal of water molecules that were already present in the hydrate. The same volume (100 µL) of Brisbane River water before filtration and after filtration was added to each LB and PDA plates. LB plates were placed in an incubator for 24 h and PDA plates were wrapped in aluminium foil and left at room temperature for 1 week. Five independent replicates were performed for each test. To quantify the bacterial removal rate under extreme conditions, 1.6 × 10^−11^ (160 billion) freshly-grown *E. coli* bacteria in 250 mL liquid LB medium were directly added onto the top of a 10 mm hydrate gel and filtrate was incubated at 37 °C on LB plates at various dilutions for 16 h before quantifying cfu.

#### Microalgal cultures used for filtration

Microalgae *Scenedesmus dimorphus* NT8c and *Haematococcus pluvialis* UQ1 were isolated from Douglas Daly Research Farm, Northern Territory, Australia and an open rooftop pond at the University of Queensland, St Lucia campus, Australia, respectively. Both cultures were grown to a density of 0.5 g dry weight L^−1^ in Bold’s Basal Medium.

#### Removal of bacteriophages by hydrate filtration

A total of 3 μL of M13K07 helper bacteriophage (10^11^ pfu mL^−1^; NEB Biolabs) was added to 20 mL of demineralized water and mixed and added to the top of a thick hydrate gel (made at 100 °C). Based on M13K07 DNA sequence, forward and reverse primers were designed. For detection by PCR, each reaction contained 7.2 μL water, 4 μL 5xHF Buffer, 1.6 μL dNTPs (10 mM), 2 μL forward, 2 μL reverse primers (10 µM each), and 0.2 μL Phusion polymerase enzyme (Thermo Scientific). A total of 3 μL of each water sample was added to the reaction mix. PCR cycling included 98 °C for 30 s, followed by 35 cycles (98 °C for 10 s, 65 °C for 30 s, and 72 °C for 30 s) and 72 °C for 10 min, and cooling to 16 °C. After PCR, 2% (w/v) agarose gel electrophoresis containing ethidium bromide was used for size separation and analysis of DNA. MS2 bacteriophages were supplied by ALS laboratories (Stafford, Brisbane, Australia). After adding 3.2 × 10^9^ pfu bacteriophages to 20 L water, it was subjected to hydrate filtration and filtrate samples were collected. The virus titer was determined as pfu by using the f-RNA phage DA-f-RNA Coliphage Double Agar Layer assay (ALS laboratories). Control measurement of the supplied concentrated phages after completion of the experiment showed no marked reduction in virus titer (trip control: 3.1 × 10^12^ pfu/100 mL).

#### Measuring turbidity, UV 254 nm and total organic carbon (TOC)

For preparing samples for turbidity, UV 254 nm and TOC measurements, turbid Brisbane River water were filtered using small filtration equipment described above (hydrate filter volume 20 mL). A portable calibrated turbidity meter (nephelometer; Lovibond, TurbiCheck) was used for measuring the turbidity in Nephelometric Turbidity Units (NTU). For measuring UV254 nm or Spectral Absorption Coefficient (SAC), a Cary® 50 Bio UV-Visible Spectrophotometer (VARIAN INC) was used. The total organic carbon (TOC) was measured using an Analytik Jena multi N/C 2100 S Total Organic Carbon Analyser.

#### Hydrate filtration of BSA protein

Crystallized bovine serum albumin (BSA; Sigma) powder was mixed with demineralized water to prepare BSA solution. A total of 50 mL of a 1 mM BSA solution was added on top of the hydrate gel.

#### Repeat use of impure hydrate gel for turbid river water filtration

Filtration of turbid river water was carried out as described above. But instead of removing the retentate from the top of the gel, all accumulated particles were repeatedly mixed together with the gel and filtration process was repeated by adding more turbid river water. A total of 50 mL of fresh gel was used for repeated filtration of 1 L portions of dirty turbid river water (OD^450 nm^ = 0.175) for each interval of filtration. The filtrate samples were collected in three replicates (at start, middle and end). To test for microbial contaminants, 100 µL of each sample were added to LB plates and incubated to quantify colony forming units as described above.

## Electronic supplementary material

Supplementary Material
