# Supplementary material for: High flux water purification using aluminium hydroxide hydrate gels
Source: Sci Rep. 2017 Dec 12;7:17437. doi: 10.1038/s41598-017-17741-z (PMC5727224; doi:10.1038/s41598-017-17741-z)
Supplement: Supplementary file 1 — Supplementary Material [file 41598_2017_17741_MOESM1_ESM.doc]

**Supplementary Material**

**Title: High flux water purification using aluminium hydroxide hydrate gels**

***Ali Malekizadeh and Peer M Schenk***

| **Supplementary Table 1. Examples of different fluxes from different membranes in comparison to aluminium hydroxide hydrate filtration.** | | | |
| --- | --- | --- | --- |
| **Filter** | **Permeate Flux (L.h^-1^,m^-2^)** | **Used for** | **References** |
| Aluminium hydroxide polyhydrate gel filtration | 28,311 at 0.8 bar  337 at 2.76 bar | Pure water filtration  Turbid water treatment | This study |
| Silicon carbide membrane (1 micron) | 8,000 at 1 bar | Pure water filtration | LiqTech International, Ballerup, Denmark |
| Poly-vinylidene  fluoride (PVDF) microfiltration (MF) | 1,600 at 1 bar | Pure water filtration | (Chae et al. 2008) |
| Millipore MF membrane (PM30) | 900-1,020 at 1 bar | Pure water filtration | (Madaeni et al. 1995) |
| Ceramic MF membrane | 600 at 1 bar | Produced water treatment | (Ebrahimi et al. 2010) |
| Inorganic metal microfiltration membranes | 180 at 0.3 bar | Surface water filtration | (Leiknes et al. 2004) |
| Silicon carbide membrane (0.01 micron) | 300 at 1 bar | Pure water filtration | LiqTech International, Ballerup, Denmark |
| Ceramic UF membrane (0.05 µm) | 150-250 at 1 bar | Produced water treatment | (Ebrahimi et al. 2010) |
| Cellulose acetate UF (Millipore Co., Bedford, MA) | 100-200 at 0.7 bar | Separation of TiO_2_  Photocatalysts in Drinking Water Treatment | (Lee et al. 2001) |
| UF membrane (A-LF) | 80-160 at 1 bar | Refinery effluent pre-treatment | (Teodosiu et al. 1999) |
| UF single fiber | 100 at 1 bar | Drinking water filtration | (Zhang et al. 2003) |
| UF | <100 | Seawater pre-treatment | (Vial and Doussau 2003) |
| UF hollow fiber  (Aquasource) | 69 at 1 bar | Drinking water filtration | (Choo et al. 2005) |
| UF, Polyethersulfone | 45 at 4 bar | meat abattoirs effluent treatment | (Cowan et al. 1992) |
| Immersed membrane bioreactor (MBR) (Zenon’s ZeeWeed® ZW-500) | 20-50 at (0.2-.5) bar | Wastewater treatment | (Cote and Thompson 2000) |
| Ceramic membrane (MBR) | 411 at 1 bar | pure water filtration | (Visvanathan et al. 2007) |
| NF 270 (Dow) | 50 at 2 bar  150 at 7 bar | DI water filtration | (Mondal and Wickramasinghe 2008) |
| NF 90 (Dow) | 12 at 2 bar  47 at 7 bar | DI water filtration | (Mondal and Wickramasinghe 2008) |
| RO, Cellulose acetate | 20-22 at 25 bar | meat abattoirs effluent after UF | (Cowan et al. 1992) |
| low pressure RO (BW30, Dow) | 12 at 2 bar  32 at 7 bar | DI water filtration | (Mondal and Wickramasinghe 2008) |
| low pressure RO (BW30, Dow) | 5 at 2 bar  8 at 7 bar | Produced water treatment | (Mondal and Wickramasinghe 2008) |

**Supplementary Figure 1 | Water-holding properties of aluminium hydroxide hydrate gels.** Aluminium hydroxide hydrate gel before (**a**) and after (**b**) drying. **c.** Assessing the evaporation rate of different aluminium hydroxide hydrates samples (2.5 mL each) over time.

**
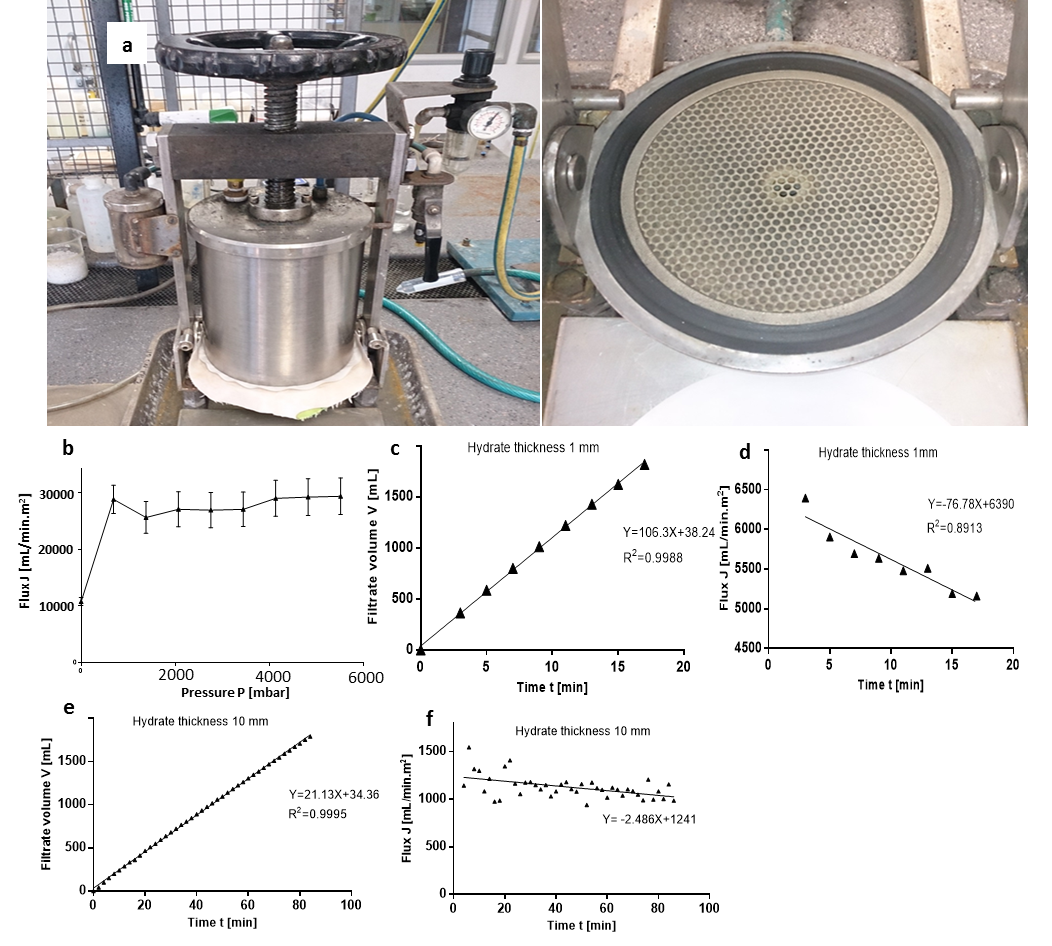
**

**Supplementary Figure 2 | Using hydrate separation for water purification. a, Pressure hydrate filtration equipment used for external pressure-assisted hydrate filtration (adjustable up to 6,9 bar (100 psig)). b. Pressure flux curve of hydrate filter. Flux was calculated in 2 min time periods, during each filtration cycle of 2 L of demineralised water (11 replicate measurements) a 1 mm gel. c. Filtrate volume change over time during river water filtration with a 1 mm hydrate filter. d. Flux change over time during river water filtration with a 1 mm hydrate filter; the average flow rate and average flux were 106.1 mL min^-1^ and 5622.1 mL m^-2^ min^-1^, respectively. e. Filtrate volume change over time during river water filtration with a 10 mm hydrate filter; the average flow rate and average flux were 21.3 mL min^-1^ and 1128.6 mL m^-2^ min^-1^, respectively (about 5 times slower than with 1 mm hydrate filter). f. Flux change over time during filtration using a 10 mm hydrate filter.**

| 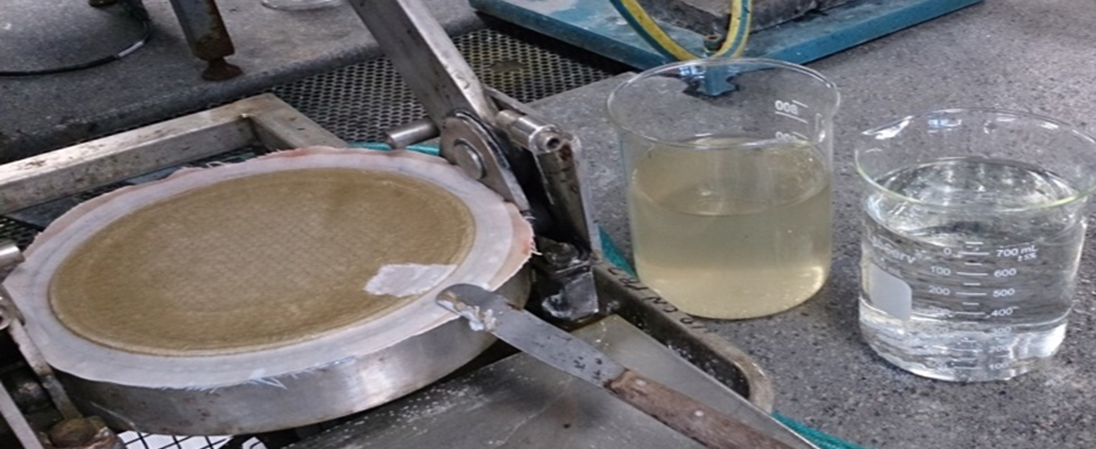  **a** |
| --- |
| 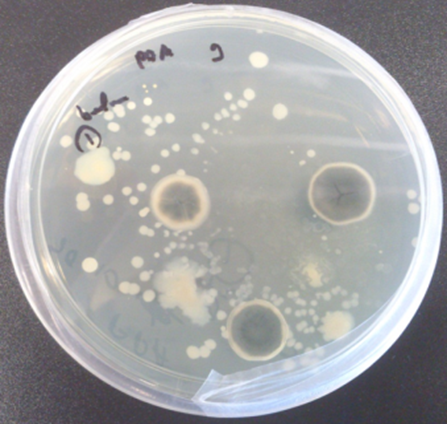 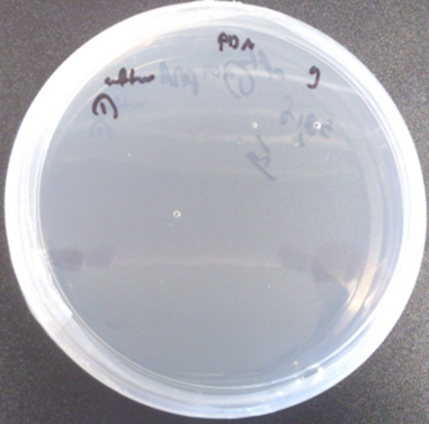  **c**  **b** |
| 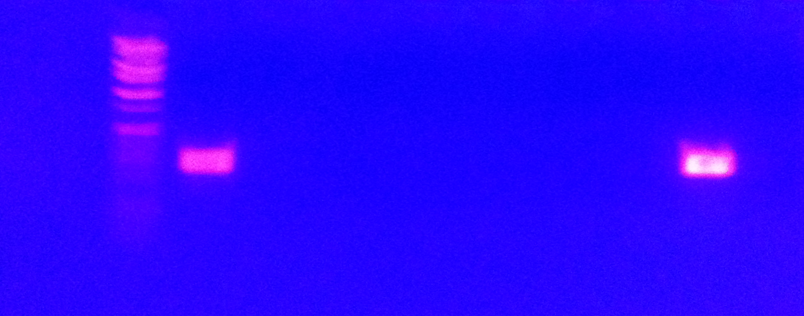  **d**  1 2 3 4 5 6 7 8 9 |
| **e**  CTTCCAGACACCGTACTTTAGTTGCATATTTAAAACATGTTGAGCTACAGCACCAGATTCAGCAATTAAGCTCTAAGCCATCCGCAAAAATGACCTCTTATCAAAAGGAGCAATTAAAGGTACTCTCTAATCCTGACCTGTTGGAGTTTGCTTCCGGTCTGGTTCGCTTTGAAGCTCGAATTAAAACGCGATATTTGAAGTCTTTCGGGCTTCCTCTTAATCTTTTTGATGCAATCCGCTTTGCTTCTGACTATAATAGTCAGGGTAAAGACCTGATTTTTGATTTATGGTCATTCTCGTTTTCTGAACTGTTTAAAGCA |

**Supplementary Figure 3 | Microbial contamination tests of Brisbane River water before and after hydrate filtration. a.** Dead end pressured hydrate filtration of Brisbane River water producing clear water. **Also s**hown are PDA plates with culturable bacteria and fungi from river water before (b) and after (**c**) filtration (after 7 days of incubation). Five independent experiments were carried out with the same result. **d.** Detection of M13K07 Helper Phage DNA following hydrate filtration. Lane 1: wide-range DNA ladder (Takara). Lanes 2 and 9: PCR-amplified phage DNA fragments as positive controls (no filtration, but phage suspension was either kept on ice (Lane 2) or at 25ºC (Lane 9) for the duration of filtration). Lanes 3-8: PCR reactions of different filtrate samples (6 independent replicates) that showed no traces of phage DNA amplification. **e.** M13K07 Helper Phage sequence with highlighted primers.

**
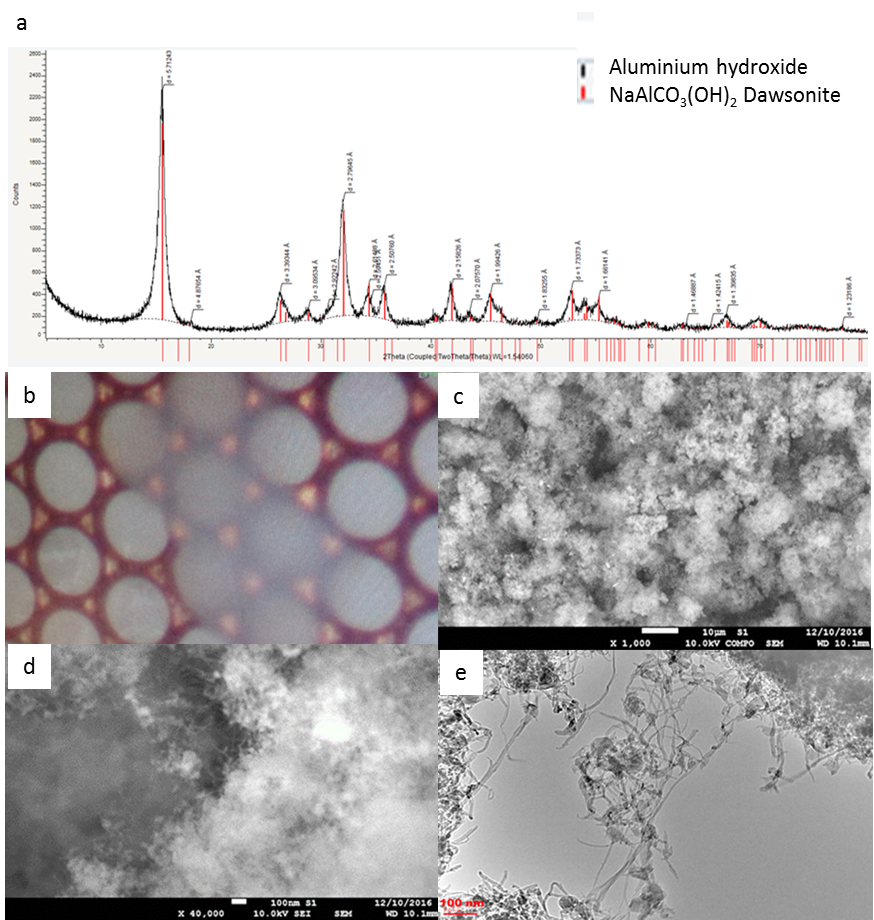
**

**Supplementary Figure 4. Material and microanalysis of dried aluminium hydroxide hydrate gel. a.** XRD data of dried aged hydrate gel identifying NaAlCO_3_(OH)_2_ (dawsonite) as the dominant phase; **b.** Aerogel sample on a TEM grid; **c-d.** Scanning Electron Microscopy (SEM) of aerogel (bar 10 µm and 100 nm, respectively); **e.** Transmission Electron Microscopy (TEM) of aerogel (bar 100 nm).


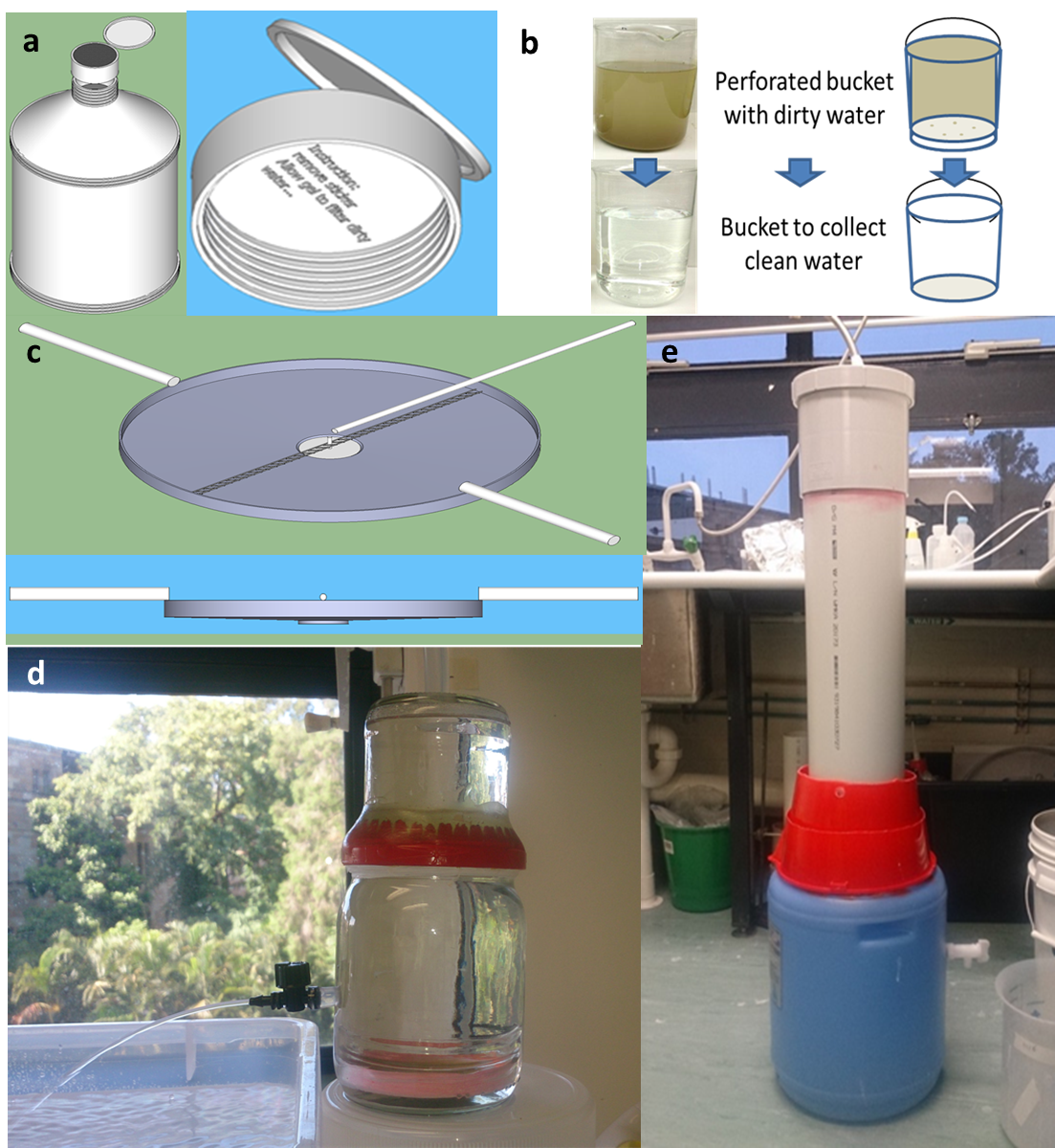


**Supplementary Figure 5. Prototypes and possible applications of aluminium hydroxide hydrate filtration a.** Design for hydrate filters in the lid of squeezable plastic water bottles. **b.** Simple two bucket purification device that could be suitable for developing countries. **c.** Perspective view of a possible design of a single-step water treatment plant. This unit may operate similarly to conventional water treatment sedimentation clarifiers, where hydrate gel filtration occurs in the middle section and conventional squeegees periodically collect the contaminants. **d.** prototype using an inverted glass jar fitted with a water inlet and a hydrate filter on a sintered titanium disc placed inside another sealed glass jar with a water outlet. **e.** Prototype using PVC pipes with the same design as (e) but at larger scale using a perforated plastic holding disc and a 2 mm hydrate layer and with direct connection to the mains water supply with a floating valve.

| 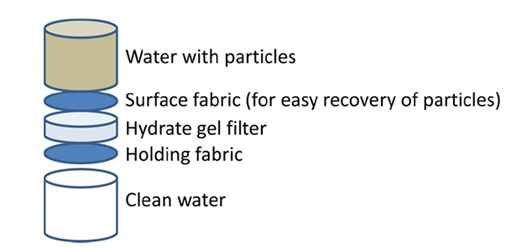  **a**  **c** | 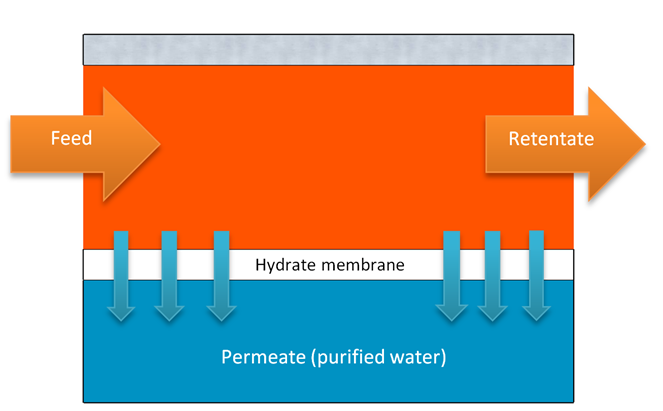  **b** |
| --- | --- |
| 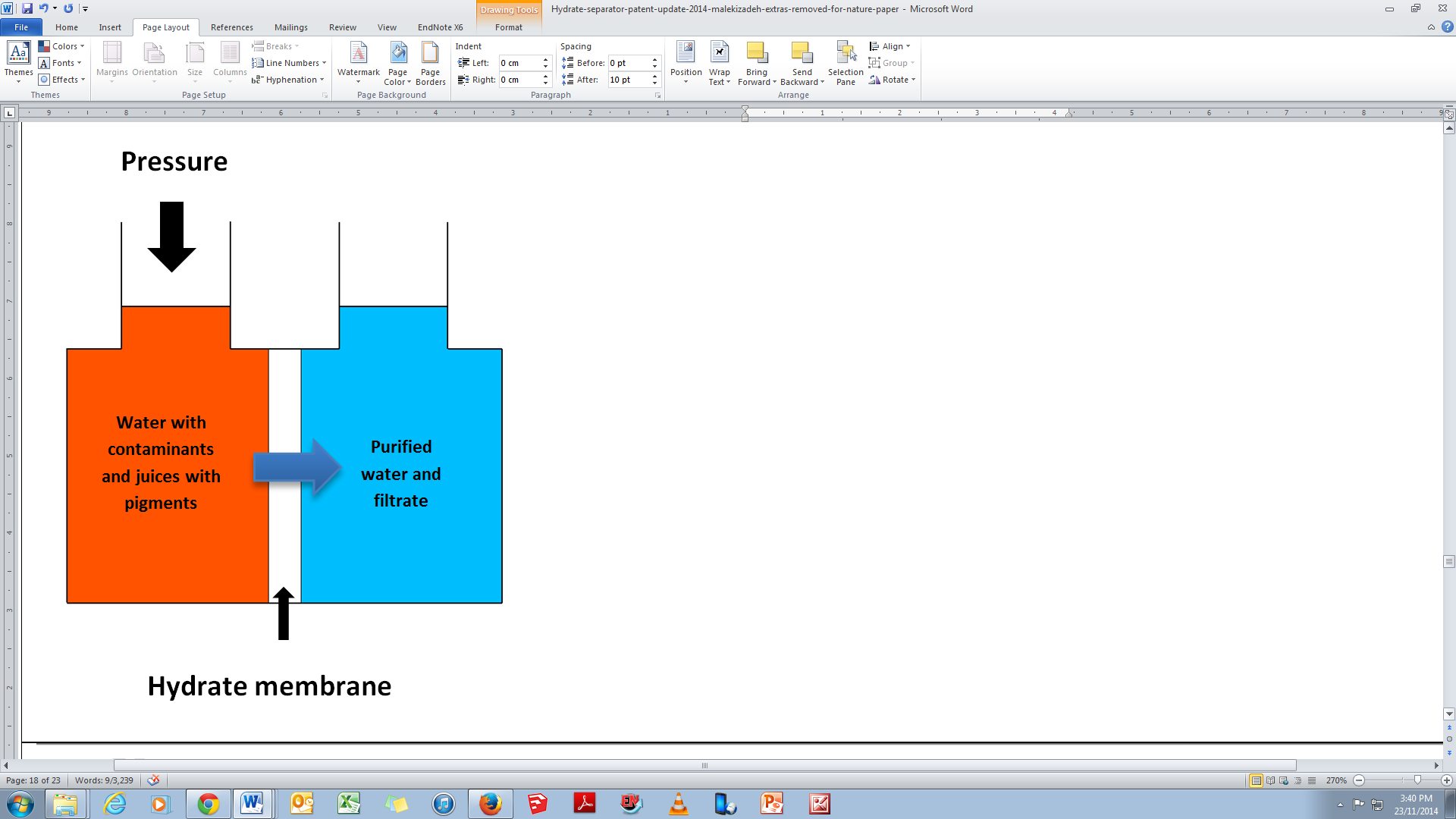 | 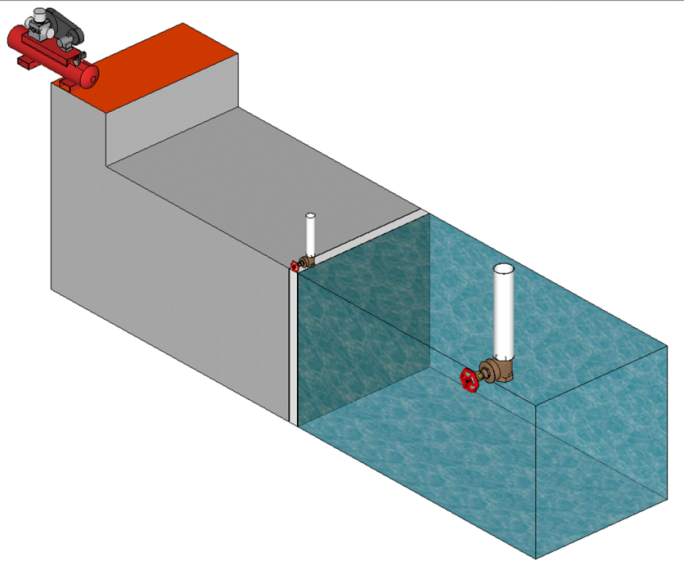  Compressor or head pressure  Tank of water with contaminations or juices with pigments  Hydrate membrane section  Purified water or filtrate  **d** |
| 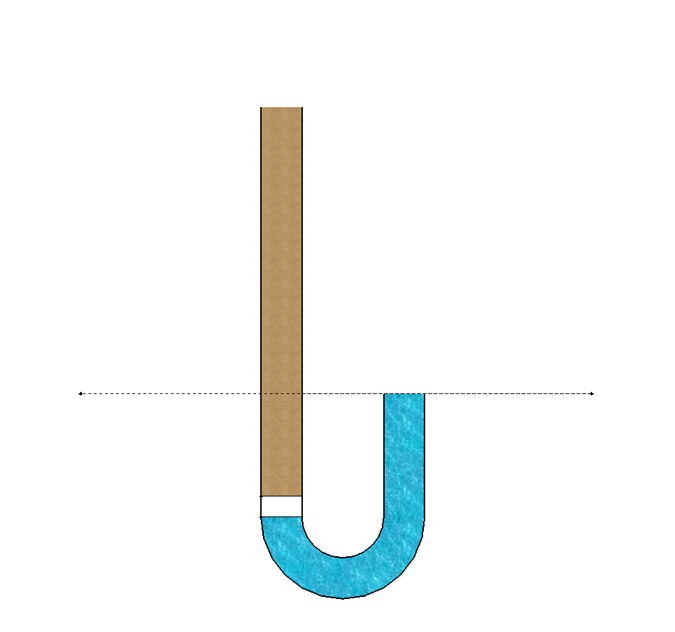  **e**  Hydrate layer  Dirty water  Clean water  1  2 | 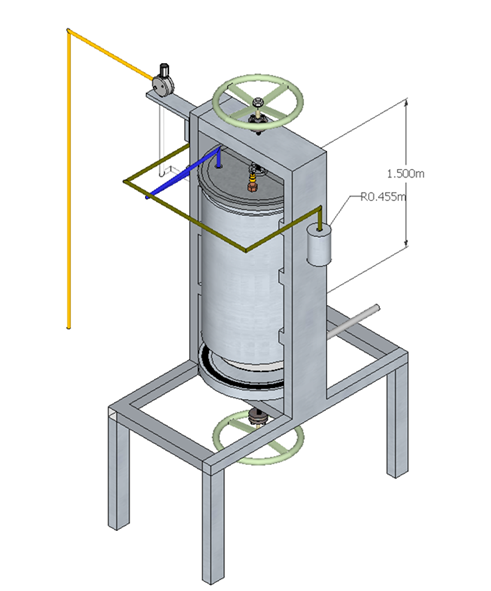  **f** |

**Supplementary Figure 6 | Possible designs of hydrate filtration devices. a.** A basic dead end design of a hydrate separator. **b.** A basic crossflow design of a hydrate separator. **c.** A basic design of a reverse osmosis-like hydrate separator. **d.** A larger scale conceptual design of the reverse osmosis-style hydrate separator design. As the hydrate filter operates relatively fast compared to conventional filters and at low pressures, even a head pressure may be enough for most applications. **e.** Another design which works similar to **c, d** but ensures that the hydrate layer always remains submerged in water to prevent drying. In this design even if the feed water stops the water level will be automatically adjust at 1. Furthermore water on top of the hydrate layer (2) protects it during refilling. **f.** Conceptual design of a dead end hydrate pressure filter unit with the cylinder welded to the stand.
